# EP80317 Restrains Inflammation and Mortality Caused by Scorpion Envenomation in Mice

**DOI:** 10.3389/fphar.2019.00171

**Published:** 2019-03-01

**Authors:** Karina F. Zoccal, Luiz G. Gardinassi, Karla C. F. Bordon, Eliane C. Arantes, Sylvie Marleau, Huy Ong, Lúcia H. Faccioli

**Affiliations:** ^1^ Departamento de Análises Clínicas, Toxicológicas e Bromatológicas, Faculdade de Ciências Farmacêuticas de Ribeirão Preto, Universidade de São Paulo, Ribeirão Preto, Brazil; ^2^ Centro Universitário Barão de Mauá, Ribeirão Preto, Brazil; ^3^ Departamento de Física e Química, Faculdade de Ciências Farmacêuticas de Ribeirão Preto, Universidade de São Paulo, Ribeirão Preto, Brazil; ^4^ Faculté de Pharmacie, Université de Montréal, Montréal, QC, Canada

**Keywords:** scorpion envenomation, EP80317, inflammation, leukotriene B_4_, mortality

## Abstract

Over 1 million cases of scorpion stings are estimated every year, whereas current treatment is limited to antivenom serum combined with supportive therapy. *Tityus serrulatus* scorpion venom (TsV) is composed of diverse molecules, including toxins that induce a catecholamine storm and mediate classical symptoms of scorpion envenomation. However, the same toxins promote an intense inflammatory response coordinated by innate immune cells, such as macrophages, contributing significantly to the lung edema and mortality caused by TsV injection. Macrophages sense TsV *via* innate immune receptors, including TLR2, TLR4, and CD14 that promote inflammation and mortality *via* PGE_2_/cAMP/PKA/NF-κB/IL-1β axis. The scavenger receptor CD36 also recognizes TsV, but in contrast to the other receptors, it drives the production of leukotriene B_4_ (LTB_4_). This lipid mediator operates *via* BLT1 receptor to reduce cAMP production and consequently IL-1β release, which results in resistance to fatal outcomes of experimental scorpion envenomation. EP80317 is an hexapeptide that serves as a ligand for CD36 and features protective effects under conditions such as atherosclerosis and vascular inflammation. In this study, we evaluated the effects of EP80317 treatment during experimental scorpion envenomation. EP80317 treatment suppressed mouse peritoneal macrophage production of IL-1β, IL-6, tumor necrosis factor (TNF-α), CCL3, and PGE_2_ *in vitro*. EP80317 treatment also boosted the production of LTB_4_ and IL-10 in response to TsV. Importantly, EP80317 restrained lung inflammation and mortality caused by TsV *in vivo*. Taken together, these data indicate a strong therapeutic potential of EP80317 as a supportive treatment to control inflammation induced by scorpion envenomation.

## Introduction

Scorpion envenomation affects more than 1 million subjects every year (Chippaux and Goyffon, 2008; Isbister and Bawaskar, 2014). In Brazil, stings by the scorpion *Tityus serrulatus* contribute significantly for this scenario, whose venom (TsV) is composed by a cocktail of bioactive compounds (Pucca et al., 2015). In recent years, it became clear that beyond the classical neuroexcitatory syndrome, inflammation also underlies the pathology and mortality caused by TsV. We and others have demonstrated that crude TsV or isolated toxins (Ts1, Ts2, Ts6) induce the production of inflammatory mediators by innate immune cells, including cytokines, nitric oxide (NO), and bioactive lipids such as prostaglandin E_2_ (PGE_2_) and leukotriene B_4_ (LTB_4_) (Petricevich, 2002; Petricevich and Lebrun, 2005; Petricevich et al., 2007; Zoccal et al., 2011, 2013). Macrophages sense TsV *via* TLR2, TLR4, or CD14, which control the release of PGE_2_ and interleukin-1β (IL-1β) (Zoccal et al., 2014, 2016, 2018b). PGE_2_ signals through EP2/4 receptors, increasing intracellular levels of cyclic adenosine monophosphate (cAMP). The cAMP-dependent protein kinase A (PKA) is activated and phosphorylates the nuclear factor-κB (NF-κB), potentiating IL-1β release (Zoccal et al., 2016). IL-1R signaling mediates activation and accumulation of neutrophils in the lung, edema, and eventually death in a mouse model of scorpion envenomation (Zoccal et al., 2016). In stark contrast to other receptors, CD36 promotes intracellular signaling that favors LTB_4_ release (Zoccal et al., 2018b). This eicosanoid reduces intracellular cAMP *via* BLT1 receptor and suppresses IL-1β production and mortality due to scorpion envenomation (Zoccal et al., 2016, 2018b). Of importance, molecular mechanisms governing responses to TsV in mouse models are strongly correlated with that of human cell responses (Zoccal et al., 2018b).

Recommended therapy is limited to antivenom serum, and depending on the severity, the serum is combined with supportive treatment such as prazosin, ionotropic agents, atropine, vasodilators, and benzodiazepines (Boyer et al., 2009; Isbister and Bawaskar, 2014). However, current therapeutic strategies do not account for tissue damage and mortality caused by TsV-induced inflammation (Zoccal et al., 2016). EP80317 is a synthetic analog of growth hormone-releasing peptides, which serves as a ligand for CD36 and exhibits cardioprotective, anti-atherosclerotic, hypocholesterolemic, and anticonvulsant effects (Marleau et al., 2005; Bujold et al., 2009, 2013; Harb et al., 2009; Bessi et al., 2012; Lucchi et al., 2017). EP80317 activates the peroxisome proliferator-activated receptor gamma (PPAR-γ) (Bujold et al., 2009), a nuclear receptor that regulates lipid bodies and lipid metabolism in response to TsV (Zoccal et al., 2015). In line with these findings, we investigated whether EP80317 limits TsV-mediated inflammation and mortality in a mouse model.

## Materials and Methods

### *Tityus serrulatus* Venom (TsV)

In accordance with the Brazilian Institute of Environment, *T. serrulatus* scorpions were maintained at the vivarium of the Ribeirão Preto Medical School, University of São Paulo. TsV was obtained by electrical stimulation, lyophilized, weighted, and stored at −80°C. Before experiments, TsV was diluted in PBS, filtered, and tested for LPS contamination as described (Zoccal et al., 2014, 2018b).

### Mice and Experimental Settings *in vivo*

All experiments using animals were approved by the Comissão de Ética no Uso de Animais da Faculdade de Ciências Farmacêuticas de Ribeirão Preto-USP (protocol #14.1.272.53.7). Six- to eight-week-old C57BL/6 female or male mice were weighed before experiments under all conditions. Animals were injected with vehicle (PBS) or a lethal (180 μg/kg, i.p.) or excessive (super dose; 360 μg/kg, i.p.) dose of TsV (Zoccal et al., 2016). Following this, TsV-injected animals were treated with vehicle or EP80317 (0.289 μmol/kg, i.p.) at 0.5 and 2 h after the venom injection (Marleau et al., 2005; Bessi et al., 2012; Bujold et al., 2013). Mice were followed during 8 h and sacrificed upon signs of severe envenomation such as difficult of breathing, unusual head, neck and eye movement, and sweating. Euthanasia was performed with an overdose of chemical anesthetics (100 mg/kg ketamine and 10 mg/kg xylazine). In one set of animals, bronchoalveolar lavage fluid (BALF) was collected for total leukocyte and neutrophil counts. In another set of animals, lungs were removed, weighted, homogenized, and stored at −80°C.

### Isolation and Treatment of Murine Peritoneal Macrophages

Resident peritoneal macrophages (PMs) were isolated from naïve C57BL/6 mice by injecting 3 ml of PBS into the abdominal cavity and massaged for 1 min. Peritoneal fluid then collected using a syringe with a needle inserted into the inguinal region as described previously (Zoccal et al., 2013). PMs (2 × 10^5^ cells/well) were cultured at 37°C, 5% CO_2_ for 2 h. After this period, PMs were incubated with EP80317 (100 nM) or vehicle (PBS) for 2 h, followed by stimulation with TsV (50 μg/ml) for 24 h at 37°C, 5% CO_2_. Culture supernatants were collected and stored at −20°C for further analysis.

### Quantification of Soluble Mediators and NF-κB Phosphorylation

Cell culture supernatants and lung homogenates were used to quantify IL-1β, IL-6, TNF-α, CCL3 (MIP-1α), and IL-10 using enzyme-linked immunosorbent assay (ELISA) kits (R&D Systems, Minneapolis, MN, United States). Quantifications of LTB_4_ and PGE_2_ were performed by enzymatic immunoassays (Enzo Life Sciences, NY, United States), after lipid extraction and purification from cell culture supernatants or lung homogenates using Sep-Pak C18 cartridges (Thermo Fisher Scientific, Bellefonte, PA, United States), as described previously (Zoccal et al., 2018b). Total protein was quantified using Coomassie Protein Assay Reagent (Pierce Chemical, Rockford, IL, United States). Nitrite (NO_2_^−^) was measured as an indicator of NO production by obtaining a standard curve using serial NaNO_2_ dilutions, as described (Zoccal et al., 2011). cAMP was quantified by ELISA, according to the manufacturer’s instructions (Enzo Life Sciences, Farmingdale, NY, United States), as described (Zoccal et al., 2018b). Cell cultures were lysed to measure phospho-NF-κB p65 (Ser536) and total NF-κB p65 using the PathScan Inflammation Multi-Target Sandwich ELISA kit (Cell Signaling, Danvers, MA, United States) as described (Zoccal et al., 2018a).

### Statistical Analyses

Data were analyzed with GraphPad Prism 5.0 software (GraphPad, San Diego, CA, United States), using one-way ANOVA followed by Bonferroni’s multi-comparison test or log-rank test. Adjusted *p* values <0.05 were considered significant.

## Results

### EP80317 Suppresses TsV-Induced Inflammation and Mortality *in vivo*

**Figure 1:**
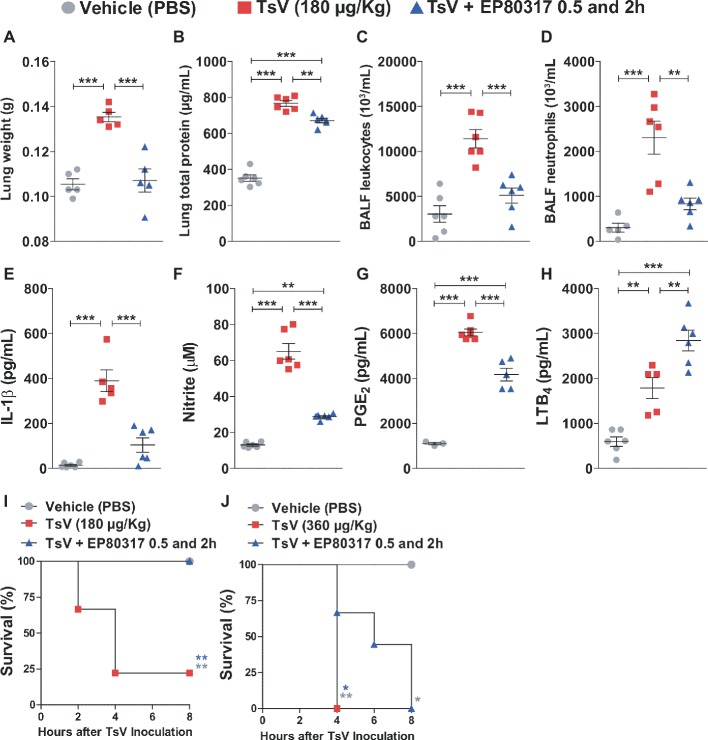
Treatment with EP80317 protects mice from scorpion envenomation. **(A–H)** C57BL/6 mice were injected with a lethal dose of TsV (180 μg/kg, i.p.) and treated with vehicle (PBS) or EP80317 (0.289 μmol/kg, i.p.) at 0.5 and 2 h after the venom injection. Lungs were removed immediately after death or at 8 h after venom injection. BALF was collected from a different set of animals under the same condition. **(A)** Lung weight, **(B)** Lung total protein concentration, (**C**) BALF total leukocyte counts, **(D)** BALF neutrophil counts**, (E)** IL-1β levels, **(F)** NO levels, **(G)** PGE_2_ levels, and **(H)** LTB_4_ levels. The experiment was conducted once with six mice per group. Differences were evaluated with one-way ANOVA followed by Bonferroni’s multi-comparison test. **(I,J)** C57BL/6 mice were injected with a **(I)** lethal (180 μg/kg, i.p.) or **(J)** excessive (superdose; 360 μg/kg, i.p.) dose of TsV and treated with vehicle (PBS) or EP80317 (0.289 μmol/kg, i.p.) 0.5 and 2 h after the venom injection. Survival was monitored for 8 h. The experiment was performed once with six mice per group, and the log-rank test was used to analyze significant differences. Data represent mean ± SDs, and significance is given by *p < 0.05, **p < 0.01, and ***p < 0.001.

EP80317 mediates protective effects *via* CD36 (Marleau et al., 2005; Bujold et al., 2009, 2013; Harb et al., 2009; Bessi et al., 2012; Lucchi et al., 2017), a scavenger receptor that drives eicosanoid metabolism toward LTB_4_ synthesis and represses inflammation and mortality caused by scorpion envenomation (Zoccal et al., 2016, 2018b). This suggests that binding of EP80317 to CD36 could modulate LTB_4_ metabolism and influence the outcome of scorpion envenomation. To test this hypothesis, C57BL/6 mice were injected with a lethal dose of TsV (180 μg/kg, i.p.) and treated with vehicle or EP80317 (0.289 μmol/kg, i.p.) at 0.5 and 2 h after the experimental envenomation. TsV injection in mice causes significant perturbations in the lung (Zoccal et al., 2016, 2018b), prompting for detailed analysis of inflammatory parameters in this organ. As expected, TsV induced an intense inflammatory response reflected by elevated lung weight (Figure 1A), total protein content (Figure 1B), accumulation of total leukocytes (Figure 1C), and neutrophils (Figure 1D), as by increased levels of IL-1β (Figure 1E), NO (Figure 1F), PGE_2_ (Figure 1G), and LTB_4_ (Figure 1H). Strikingly, treatment with EP80317 significantly impacted the inflammatory response, promoting further increase of LTB_4_ levels (Figure 1H) while reducing all other parameters (Figures 1A–G). These results suggest that EP80317 might restrain the inflammation-mediated mortality induced by TsV. To address this question, C57BL/6 mice were injected with a lethal (180 μg/kg, i.p.) or an excessive (super dose; 360 μg/kg, i.p.) dose of TsV and later treated with vehicle or EP80317 (0.289 μmol/kg, i.p.) at 0.5 and 2 h after the experimental envenomation. We observed 75% mortality of mice injected with a lethal dose of TsV, whereas 100% of mice survived after treatment with EP80317 (Figure 1I). Moreover, excessive TsV dose induced 100% of mortality, independently of EP80317 treatment (Figure 1J). However, non-treated mice died within 4 h of envenomation, while EP80317 significantly extended mice survival.

### EP80317 Modulates Inflammatory Pathways in Macrophages Activated by TsV

**Figure 2:**
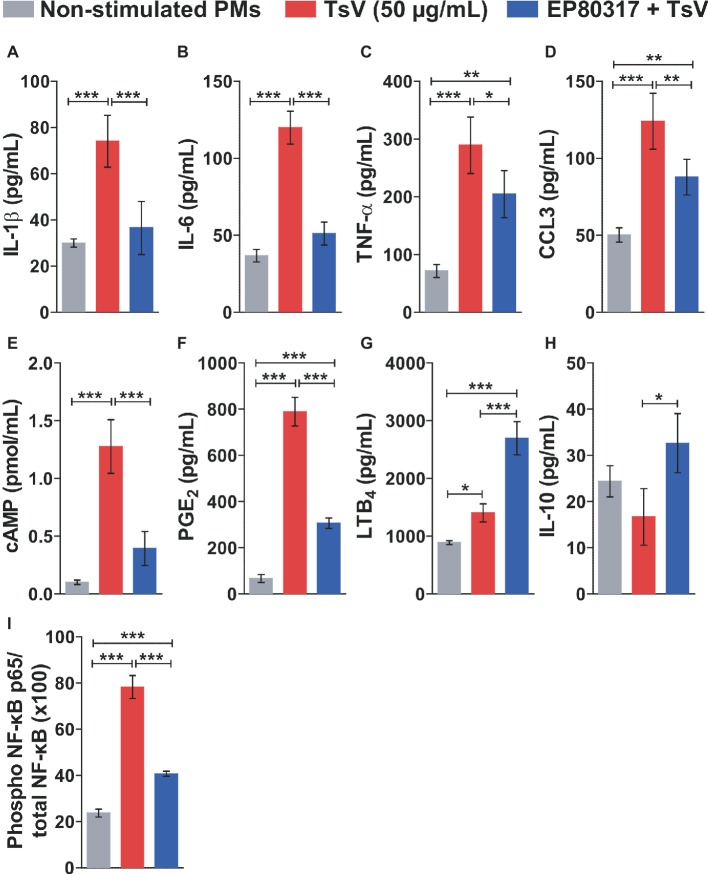
EP80317 inhibits NF-κB activation and production of inflammatory mediators in TsV-stimulated macrophages. **(A–H)** Peritoneal macrophages (PMs) were incubated or not with EP80317 (100 nM) for 2 h and later stimulated with TsV (50 μg/ml) for 24 h for quantification of cytokines/chemokine, or 5 min. for quantification of cAMP. **(A)** IL-1β, **(B)** IL-6, **(C)** TNF-α, **(D)** CCL3, **(E)** cAMP, **(F)** PGE_2_, **(G)** LTB_4_, and **(H)** IL-10. **(I)** PMs were pre-treated or not with EP80317 (100 nM) for 2 h and then stimulated with TsV (50 μg/ml). Cell lysates were obtained 2 h later for phospho-NF-κB p65 (Ser536) and total NF-κB p65 quantification. Data represent one of two independent experiments (*n* = 4 replicates). Differences were evaluated with one-way ANOVA followed by Bonferroni’s multi-comparison test. Data represent mean ± SDs, and significance is given by *p < 0.05, **p < 0.01, and ***p < 0.001.

To determine the molecular mechanisms by which EP80317 restrains inflammation and mortality induced by TsV, peritoneal macrophages were isolated from C57BL/6 mice and incubated or not with EP80317 for 2 h. Following, macrophages were stimulated with TsV for 24 h in the presence of EP80317 throughout the challenge. Macrophages stimulated with TsV produced increased levels of the pro-inflammatory cytokines IL-1β (Figure 2A), IL-6 (Figure 2B), and TNF-α (Figure 2C); the chemokine CCL3 (Figure 2D); the second messenger cAMP (Figure 2E); and the bioactive lipids PGE_2_ (Figure 2F) and LTB_4_ (Figure 2G). Of note, exposure to EP80317 potentiated LTB_4_ synthesis (Figure 2G) and induced the production of the anti-inflammatory cytokine IL-10 (Figure 2H). At the same time, EP80317 abrogated the production of IL-1β (Figure 2A), IL-6 (Figure 2B), and cAMP (Figure 2E) and significantly reduced levels of TNF-α (Figure 2C), CCL3 (Figure 2D), and PGE_2_ (Figure 2F). The transcription factor NF-κB targets genes coding for these cytokines, chemokine, or even the rate-limiting enzyme involved in PGE_2_ synthesis, cyclooxygenase-2 (COX-2). This suggests that EP80317 might restrain the production of inflammatory mediators by suppressing NF-κB activity. As expected, exposure to EP80317 significantly reduced NF-κB phosphorylation induced by TsV stimulation (Figure 2I).

## Discussion

Here, we explored the therapeutic potential of the CD36 ligand, EP80317, for the control of inflammation-dependent mortality caused by scorpion envenomation. Overall, data point to a model of action in which EP80317 interacts with CD36 and dampens TsV-mediated inflammation *via* molecular mechanisms that are at least partially dependent on LTB_4_ production *in vitro* and *in vivo*. Signaling *via* BLT1 receptor inhibits cAMP synthesis and reduces the activation of PKA, consequently diminishing NF-κB phosphorylation and activation (Zoccal et al., 2016). This results in reduced levels of diverse inflammatory mediators, including IL-1β. EP80317 induces intracellular increase of 15-deoxy-delta(12,14)-prostaglandin J_2_ (15d-PGJ_2_) *via* COX-2 pathway in macrophages (Bujold et al., 2009). PPAR-γ negatively regulates NF-κB in macrophages stimulated with TsV (Zoccal et al., 2015), while 15d-PGJ_2_ activates this transcription factor and might contribute to reduced inflammation. PGE_2_ and the precursor of 15d-PGJ_2_, PGD_2_, exhibit opposite effects on macrophages (Pereira et al., 2018). Thus, PGD_2_ could also play a significant role in this process by downregulating cAMP levels *via* DP2 receptor and influence NF-κB activity. Taken together, these data provide important insights into operating molecular mechanisms and potential supportive therapy with EP80317. It also encourages further investigation during envenomation by other scorpions or even other poisonous animals, since similar molecular mechanisms take place upon stimulation of cells with venoms from two species of *Bothrops* snakes (Zoccal et al., 2018a).

Interestingly, CD36 activates the p130Cas-binding kinase Pyk2 in macrophages stimulated with oxidized phospholipid, whereas previous exposure to EP80317 inhibits Pyk2 phosphorylation (Harb et al., 2009). This correlated with reduced levels of plasma IL-6, as well as reduced expression of NADPH oxidase, inducible nitric oxide synthase and CCL2 in the vasculature of apolipoprotein E-deficient mice fed a high-fat, high-cholesterol diet, and treated daily with EP80317 (Harb et al., 2009). Although we have observed that EP80317 promotes LTB_4_ synthesis or that CD36 deficiency abrogates the production of this lipid mediator in response to TsV (Zoccal et al., 2018b), the signaling pathways leading to this phenomenon are unknown. Furthermore, we observed previously that CD36 deficiency increases IL-1β release by macrophages stimulated with TsV, but cytokines such as IL-6 and TNF-α were significantly reduced (Zoccal et al., 2018b). With exception of LTB_4_ and IL-10, treatment with EP80317 significantly reduced all the evaluated inflammatory parameters, suggesting additional anti-inflammatory mechanisms. Indeed, EP80317 could induce TLR heterodimer complex dissociation and suppress TLR-mediated signaling (Triantafilou et al., 2006; Stewart et al., 2010). However, TLR2 deficiency enhances PGE_2_ production by macrophages upon TsV stimulation (Zoccal et al., 2018b). Beneficial inhibition of the immune response during scorpion envenomation is further supported by the fact that dexamethasone, a potent immunosuppressive compound, completely abrogates IL-1β release by human peripheral blood mononuclear cells in response to TsV (Zoccal et al., 2018b). Collectively, these studies provide a proof of concept for the therapeutic potential of EP80317 during scorpion envenomation, involving multiple signaling pathways in mitigating inflammation induced by TsV.

## Data Availability

All datasets generated for this study are included in the manuscript and/or the supplementary files.

## Ethics Statement

All experiments using animals were approved by the Comissão de Ética no Uso de Animais da Faculdade de Ciências Farmacêuticas de Ribeirão Preto-USP (protocol 14.1.272.53.7) and carried out in accordance with the ethical principles for animal research adopted by the Sociedade Brasileira de Ciência em Animais de Laboratório.

## Author Contributions

KZ designed and performed the experiments. KZ and LG analyzed the data and wrote the manuscript. EA and KB provided the scorpion venom. EA, KB, SM, and HO discussed the data. SM and HO provided the EP80317. LF conceived and supervised the study. All authors read and approved the final manuscript.

### Conflict of Interest Statement

The authors declare that the research was conducted in the absence of any commercial or financial relationships that could be construed as a potential conflict of interest.
